# Understanding prosocial behaviour: perspectives from different cultures and generations

**DOI:** 10.1080/00049530.2025.2507634

**Published:** 2025-05-26

**Authors:** Berlian G. Septarini, L. J. Breen, T. Hamamura

**Affiliations:** a Faculty of Psychology, Airlangga University, Surabaya, Indonesia; b School of Population Health, Curtin University, Perth, Australia

**Keywords:** Prosocial behaviour, generational differences, cultural differences, Indonesia, Australia

## Abstract

**Objective:**

Prosocial behaviour is essential for human existence such that it is expected in every society. However, different pathways of social change experienced by different societies over time may implicate different ways in which prosocial behaviour is understood and experienced. The current study aims to understand how societies experiencing different contexts of social changes hold their perspectives towards the meaning and practices of prosocial behaviour.

**Method:**

A total of 42 participants from Indonesian and Australian younger and older generations participated in a focus group designed to explore cultural and generational diversity in prosociality. Themes were generated using thematic analysis and the multiple meaning of the data were discussed using a consensual qualitative research-modified (CQR-M) approach.

**Results:**

Four themes and 23 subthemes were identified in the dataset. Findings from this study suggest that forms of prosocial behaviours are similar across cultures and generations. However, cultural differences observed in the older and younger generations reflect that Australia and Indonesia may hold different perspectives of prosocial behaviour. Interestingly, generational differences were evident in Indonesian but not in Australian participants.

**Conclusions:**

The current study suggests that understanding and experience of prosocial behaviour are not congruent between societies going through different experiences of sociocultural changes.

## Key points

What is already known about this topic:

(1) The theory of social change explains the predominant direction of changing value orientation from collectivism to individualism which affect behavioural-level changes into more self-centred behaviours such as competition rather than cooperation.

(2) Prosocial behaviour may reflect societal and individual-level changes in worldview from the self-centred to the service-oriented mode.

What this topic adds:

(1) Some understanding and experiences of prosocial behaviour were similar across four groups of participants recruited in Indonesia and Australia.

(2) Decisions to act prosocially differed between Indonesian and Australian older adults with responses ranging from intuitive to deliberative process of thoughts. For younger adults, cultural variations were evident in terms of the presence of prosocial motives to reduce aversive arousal and targeting underprivileged groups as prosocial recipients.

(3) Across cultures and generations, empathic concern or sympathy as the motive of prosocial behaviour differed.

(4) Evidence of generational differences in prosocial behaviour views was found among Indonesian participants but not Australian participants, indicating potentially less congruent views between age groups in Indonesia than in Australia.

## Introduction

Prosocial behaviour refers to positive behaviours that are intentionally and voluntarily directed to the enhancement of others’ well-being for which the motive is unspecified (Eisenberg, 2003). Prosocial behaviour is the essence of human psychological functioning which is fundamental for human existence (Schroeder & Graziano, 2017). According to Twenge et al. (2007), prosocial behaviour is “vital to the social system” (p. 56) because by acting prosocially, individuals conform with the social norm and identify themselves as part of the group, receive the benefits of being a member of the group (e.g., fulfilment of social needs), and in return, affirm membership of their group. Therefore, prosocial behaviour is expected in every society for the reason of maintaining the presence of the group over time.

Prosocial behaviour is an important field in psychology research deserving much attention. However, despite the rapid progress in prosocial behaviour research, the concept of prosocial behaviour remains obscured (Bierhoff, 2002; Gilbert et al., 2019). In particular, prosocial behaviour may be defined and performed differently across cultures (Callaghan & Corbit, 2018) such that cultural contexts should be accounted for in understanding prosocial behaviour. The way society conceptualises prosocial behaviour may vary from another since culture represents a shared understanding of words, meanings, and sample behaviours. Additionally, because changes in ecological, cultural, societal, and behavioural characteristics are interconnected, societal goals and group norms that shape prosocial behaviour may differ due to changes in these characteristics over time (Greenfield, 2016; Varnum & Grossmann, 2017). As such, stability and changes in prosocial behaviour should be understood not only in different cultural settings but also within the context of changing sociocultural environment.

A literature review of research examining prosocial behaviour in Indonesia and Australia found that prosocial behaviour had been investigated using different perspectives with various types and conceptualisations (Septarini, 2023). This finding is consistent with the assertion that prosocial behaviour may be understood differently depending on what, when, and where a society judges whether an action can be defined as prosocial. The concept of worldview also contributes for understanding variations in prosocial behaviour as the result of their interpretation of the past, present and future, as individuals in each culture may formulate their own meaning to understand prosocial acts (Markus & Kitayama, 1991; Schlitz et al., 2010). The way prosocial behaviour is understood and practised among people across cultures over time is crucial for understanding the transformation or maintenance of such behaviour within societies. A qualitative approach, which is rarely used to examine this issue (Septarini, 2023), is suitable to understand perspectives from people of different cultural and generational backgrounds towards the meaning and practices of prosocial behaviour.

### Social change, cultural dynamics and prosocial behaviour

Societies may differ in judging what is prosocial depending on historical, economic, and sociocultural circumstances such that prosocial behaviour may be understood differently across cultures (Dovidio et al., 2017). In considering the possible cross-cultural variability in prosocial behaviour, one useful theory is Greenfield’s multilevel theory of social change and human development. Greenfield (2016) proposed that changes in sociodemographic characteristics implicate changes in behaviour level. For example, sociodemographic changes from preferences to living with others to living alone have directed the shift of the learning environment/socialization practices (i.e., from social guidance to independent learning). At the individual behavioural level, an increased tendency to favour competition rather than cooperation can be expected within the society following this direction of value change. Although changes at individual behavioural level may not be simply aggregate to changes at sociodemographic level, Greenfield’s (2016) perspective is useful as a framework where behavioural changes are understood as shaped by multiple layers of contextual factors. In line with this assertion, stability or changes in prosociality may be understood through research within the context of sociocultural changes.

Insights and observations about sociocultural changes available in Greenfield’s theory are more formally articulated in the perspective of cultural dynamics (Kashima, 2014). Cultural dynamics theory holds the view that culture represents both stability and flexibility. In this perspective, prosocial behaviour can be regarded as cultural information subject to transmission that can be formed, changed, and maintained over time (Kashima, 2001). Therefore, prosocial behaviour may not only change in response to sociodemographic changes, but it may also be maintained by the society through mechanisms that transmit cultural information over generations. In fact, it is understood that cultural transmission is “the heart of the mechanism” (Kashima, 2016, p. 93) which play a central role in the maintenance of behavioural norms within societies (Schönpflug & Bilz, 2009). Cultural transmissions may occur horizontally from peers, vertically from parent to child, or obliquely from an older generation to a younger generation.

In considering patterns of sociocultural change, it is worth noting that previous literatures have suggested the direction of social change to increased individualism rather than collectivism (Greenfield, 2016; Santos et al., 2017). This line of work suggests that individualism is increasing around the world and that this may be related to changes in prosocial behaviour. Smith (2019) highlighted that the trend in increasing individualism and decreasing collectivism may reflect stronger “emancipative values” (p. 1197) which affirm autonomy, equality, and tolerance. In fact, using the data from 24 countries between 2009 and 2014, Smith (2019) concluded that increases in prosocial behaviour (i.e., helping, donating, and volunteering) were more frequent in collectivist countries such that collectivism is a strong predictor for prosocial acts. Interestingly, Luria et al. (2015) found that individualism-collectivism provides more direct prediction towards prosocial behaviour than other dimensions of culture such as power distance and uncertainty avoidance. Taken together, the literatures suggest the importance of individualism-collectivism dimension, which is strongly linked to sociodemographic changes (Greenfield, 2016; Santos et al., 2017), in explaining prosocial behaviours.

### A comparison of Australia and Indonesia

To understand cultural and generational perspective on prosocial behaviour, the current study focuses on two distinctive cultural and generational samples that appear to hold different constructions of worldview. Previous cross-cultural studies compared Australia and Indonesia on individualism-collectivism reported that Australia has been firstly identified as an individualist, whereas Indonesia predominantly shares collectivistic values in its society (Irwin, 2009). According to Triandis et al. (1986), Indonesia placed family integrity and interdependence as highly important, whereas the value of self-reliance and hedonism were placed in the lowest priority. However, following the global trends towards rising individualism, a recent report showed that Indonesia is currently transitioning to an individualistic society as indicated by smaller household size, a higher percentage of people living alone, and a higher ratio of divorces (Santos et al., 2017).

Prosocial behaviours are also changing in Australia and Indonesia. The CAF World Giving Index provides the annual report of giving behaviour across the world. In 2018, the survey reported three types of giving behaviours (i.e., helping strangers, charity donation and volunteering) between 2013 and 2017 from 146 countries. According to this report, despite the similar proportion of people engaging in giving behaviour reported in Indonesia and Australia (59%), proportions of the preferred types of giving changed. In Indonesia, donating (78%) and volunteering (53%) were more prevalent, whereas, in Australia, helping a stranger (65%) and donating (71%) were more frequently reported. Smith (2019, p. 1204) reported that between 2009 and 2016 one of the largest increases in the proportion of respondents donating was reported in Indonesia. He further noted that both value changes (collectivism-individualism) and specific events (refugee problems, political tensions, natural disasters) might be responsible for changes in donating. Taken together, the current literature suggests that prosocial behaviour may be undergoing changes in Australia and Indonesia.

Social changes experienced by individuals in Australia may not be as divergent as found in Indonesia which presumably is experiencing transformative changes in cultural values and orientations (from collectivist to more individualist). Therefore, conceptions and practices of prosocial behaviour found among different generations of Indonesian adults may not as congruent as found among different generations of Australian adults.

## Aims of study

This study aims to understand the meaning and practices of prosocial behaviour among younger and older adults in Australia and Indonesia. In particular, this study was designed to interpret the underlying meaning of prosocial behaviour in Indonesia and Australia: conceptions, types, and motives that may contribute to the maintenance of prosocial acts within societies. The research question is: how do people in Indonesia and Australia construct the meaning and practises of prosocial behaviour? Findings from this study offers foundation for future study investigating formulation, change, and maintenance of prosocial behaviour as a form of cultural information over time within societies.

## Methods and materials

The current study used an exploratory, qualitative research design to explore cultural variations of prosocial behaviour. An interpretivist approach was taken to understand the multiple meanings of phenomena (Creswell & Creswell, 2018). Gilbert et al. (2019) suggested that only through discussion could one gain a shared understanding of a particular construct. Therefore, focus groups were utilised to provide natural settings for participants to interact with each other (Braun & Clarke, 2013). Additionally, the use of focus groups allows the participants to formulate shared meanings of the topic being discussed in their everyday language, rather than from the theoretical perspective of the researcher (Bloor, 2001). Data were collected from multiple focus group discussions involving participants from four types of groups representing different cultures and generations (Australian younger adults, Australian older adults, Indonesian younger adults, and Indonesian older adults). Participants were encouraged to elaborate, exchange, and negotiate their thoughts with others to seek a collective understanding of prosocial behaviour. As such, diverse perspectives were generated to better understand the construct of prosocial behaviour across cultures and generations.

### Participants

Following ethics approval, participants were recruited in each country across age categories. In each country, all participants self-identified as either Australian or Indonesian citizens with English or Bahasa Indonesia being their first language, aged between 17 and 26 (younger generation) or 38 to 55 (older generation), and were living in urban areas (Perth metropolitan area for Australian groups and Surabaya City for Indonesian groups). People who do not use English or Bahasa Indonesia as the 1st language were not recruited into the study to limit participants from different ethnic and racial backgrounds beyond the inclusion criteria. The two age groups were selected to enable generational variability in constructing the meaning and practices of prosocial behaviours within each cultural sample. The primary targeted age group was young adults (age 17 to 26) which has been argued to have a higher probability of engaging in various prosocial activities (Carlo & Randall, 2002; Knafo & Schwartz, 2008). To contrast between generations, older generation (age group 38 to 55) was selected. Inclusion criteria were consistent to optimise group comparability across cultures. There were a total of 42 participants across seven focus groups. The group size was kept small (from 4 to 8 participants) to facilitate discussion with sufficient time for considerable input from each member yet adequate to yield variability in information (Bloor, 2001).

#### Australian older adults

Eight Australian older adults participated in a focus group discussion (*N* = 8, 87.50% female, age range = 38–50, *M* = 44.88). Participants have stayed in Perth metro area at the average of 24.38 years, 62.50% of them hold a minimum of undergraduate qualifications. Half of the participants were employed, and the other half were currently enrolled as full-time university students.

#### Australian young adults

Two focus groups were conducted for Australian young adults. The first group comprised eight participants and the second had four (*N* = 12, 75% female, age range = 17–25, *M* = 21.33). Participants have stayed in Perth metro area for about 15.50 years, 41.67% of them hold a minimum of undergraduate qualifications. Two-thirds (66.67%) were full-time university students and one-third (33.33%) were employed.

#### Indonesian older adults

Focus group data were collected from two groups of Indonesian older adults (*N* = 12, 66.67% female, age range = 38–53, *M* = 44.92). Participants have stayed in Surabaya city for about 33.17 years, 70.00% of them hold a minimum of undergraduate qualifications. More than half of the participants were employees (66.67% employees, 33.33% unemployed).

#### Indonesian young adults

Focus group data were collected from two groups of Indonesian young adults (*N* = 10, 70% female, age range = 22–26, *M* = 23.40). Participants have stayed in Surabaya city for about 8.15 years, 75.00% of them hold a minimum of undergraduate qualifications. Each group initially had six sign-ups, yet only five participants attended the session. The Indonesian young adults were mainly full-time university students (60% students, 30% employees, and 10% unemployed).

### Focus group protocols

A focus group protocol on English was developed by a team of researchers. The protocol was back-translated to ensure construct equivalence in Bahasa Indonesia (Behling & Law, 2000). Each session was designed to take up to 90 minutes, with a 15-minute group introduction. The core session was designed to take an hour and centred on four main questions formulated to address the study objectives: “what kind of positive behaviour is done intentionally and voluntarily to benefit others?”; “what makes people decide to take that kind of action?”; “to who are these positive behaviours directed?”; and “what is the impact of performing such behaviour?”. Each session closed with a 15-minute summary of the discussion, and participants were afforded the opportunity to provide additional comments.

Two volunteers with English as their first language and experienced in delivering focus group discussions facilitated the discussion with the Australian groups. One facilitator was a woman with a postgraduate qualification and was employed in a student support role within a university. The second facilitator was a man with a doctoral qualification who managed a university learning centre. Prior to data collection, the first author and both facilitators discussed and practised the protocol to ensure all questions had been asked without potential bias. Throughout the process of data collection, the first author observed the groups and took notes. The Indonesian focus groups were facilitated by the first author, who is proficient in Bahasa Indonesia and followed the same protocol used for Australian groups.

### Procedure

The study adopted a purposeful sampling strategy (Clark & Creswell, 2014) by applying particular criteria (i.e., country and age categories) so that participants in each group shared similar demographic characteristics. Recruitment flyers were distributed in public places and advertised on university official communication platforms. Additionally, some Australian participants were recruited through the university research participant pool. Potential participants were provided with an informed consent sheet and asked to indicate their availability. At the end of each focus group session, participants were offered a voucher or credit points (for student participants recruited through the research participant pool) for their time attending the session. The group discussions were audio-recorded and transcribed in the participants’ original language. The first author made transcriptions for Indonesian groups. For Australian data, transcriptions were provided through a paid service.

Data from the Indonesian groups were kept in the original language for the subsequent analysis. The challenge of using conventional translation in cross-cultural research has been criticised for inadequate conceptual congruence (Larkin et al., 2007). They argued that the predominantly translation procedure has overpowered word equivalence rather than conceptual equivalence such that the capability of each language to explain its own meaning became declined. Therefore, keeping the qualitative data in its original language provides support for the source language in generating meanings as naturally as it should. The English translation was used for Indonesian data only when labelling the codes and synthesising the codes into themes to enable comparison across groups.

### Data analysis

Thematic analysis (Braun & Clarke, 2013) was used to allow the flexible approach to identifying themes that are data-driven and theory-driven within analysis. Six overlapping analytic steps were followed, starting with reading and re-reading the transcription to familiarise the data. The second step involved coding the data using NVivo 12. All data extract were coded by the first author. For Indonesian data, extracts that contained codes were translated by the first author into English such that labels can be generated in English as well. Codes with similar patterns and meanings were collated under a theme in the third step. In this stage, codes were scrutinised to review connections with each other and identify central concepts that capture similar ideas. The fourth step involved reviewing these concepts by the research team to identify potential overarching themes and subthemes by exploring relationships between them. Themes and subthemes were then defined and named in the fifth step. At this stage, the existing literature on types (Carlo & Randall, 2002) and motives (Eisenberg et al., 2016) of prosocial behaviour were consulted to label the themes. The analysis was then finalised by producing the report.

To explore generational and cultural differences across groups, the study adopted consensual qualitative research – modified (CQR-M) as suggested by Spangler et al. (2012). CQR-M is a useful tool for the exploration and discovery of phenomena that accommodates comparison using large participants with simple qualitative data. This approach was used to determine the prevalence of themes within each group by establishing consensus within the research team. Any disagreements within the research team regarding the prevalence of the themes across groups were discussed to seek mutual understanding. Within each group, the extent to which a theme was prevalence was categorised into four types: (1) general, if a theme was present to all or all but one of the participants; (2) typical, if a theme applied to more than half of the participants; (3) variant, if a theme applied to less than half of the participants; and (4) absent, if the theme does not present in a group. For example, the subtheme “How people decide to act prosocially” was mentioned by more than half (*n* = 9) of the participants in the Australian young adult group (*N* = 12) such that the prevalence of this subtheme was “Typical” for this group. Following Ladany et al. (2012), differences between generations (younger and older) and cultures (Australia and Indonesia) were noted when there was a two-category discrepancy or more for each theme and subtheme (i.e., absent vs. typical; variant vs. general; and absent vs. general).

### Trustworthiness

The study described the specific context and circumstances of how the research was undertaken in Indonesia and Australia to enhance the credibility of the study. The primary analysis was conducted by the researchers, who kept a detailed audit trail of all actions and analytic decisions. Participants had the option to confirm, add to, or challenge the data summary presented in detail near the end of each focus group, which is an appropriate form of participant validation for focus groups (Bloor, 2001). Team members examined the draft analyses to review and discuss the coding scheme. The engagement in these processes minimised researcher bias and promoted rigour in the extraction and development of data themes. The thematic analysis was conducted following the 15-point criteria for good thematic analysis (Braun & Clarke, 2013). To ensure the study had been reported comprehensively, the 32-item COREQ checklist (Tong et al., 2007) was followed. The study used triangulation via researchers (Braun & Clarke, 2013) by involving a team of researchers in data collection and analysis.

## Results

Participants’ perspectives towards prosocial behaviour were organised in four themes: (a) layperson’s understanding of prosocial behaviour; (b) types of prosocial behaviour; (c) prosocial motivations, and (d) recipients of prosocial behaviour. Table 1 lists the themes and subthemes that we developed as well as their prevalence within each of the four groups. Examples from extracts for each theme and subthemes are provided in the Appendix.

**Table 1.** Summary of themes and subthemes representing participants’ perspective towards prosocial behaviour.

Themes and subthemes		Occurrence/Commonality of a theme^*)^				Group Difference^**)^
		Australian Group		Indonesian Group		
		Older Adult	Young Adult	Older Adult	Young Adult	
***Theme 1: Layperson’s understanding of prosocial behaviour***						
1.1	What it means to be prosocial	Variant	Variant	Variant	Variant	None
1.2	How people decide to act prosocially	Variant	Typical	General	Typical	Cultural differences among older adults
1.3	The predictors of prosocial acts	Typical	Typical	Typical	Variant	None
1.4	The changing nature of prosocial behaviour	Variant	Typical	Variant	Variant	None
***Theme 2: Types of prosocial behaviours***						
2.1	Altruistic prosocial behaviour	Variant	Variant	Typical	Typical	None
2.2	Anonymous prosocial behaviour	***absent***	Variant	***absent***	Variant	None
2.3	Prosocial behaviour for compliance	Typical	Typical	Variant	Typical	None
2.4	Prosocial behaviour in dire circumstances	Variant	Variant	Variant	Variant	None
2.5	Public prosocial behaviour	Typical	Variant	Variant	Variant	None
***Theme 3: Prosocial motivations***						
3.1	Empathic concern or sympathy	General	Typical	Variant	General	Generational differences among Indonesian samples & Cultural differences among older adults
3.2	Adherence to internalised principles	Variant	Variant	Variant	Variant	None
3.3	Adherence to social norms	Typical	Typical	Variant	Variant	None
3.4	Empathic joy and sustaining positive emotion	Typical	Typical	Variant	Typical	None
3.5	Social relatedness	Variant	Variant	Variant	Variant	None
3.6	Goal completion and increased feelings of competence	Variant	Typical	Variant	Typical	None
3.7	Reduce aversive or negative arousal	Variant	Typical	***absent***	***absent***	Cultural differences among younger adults
3.8	Social approval and reputation-related rewards	***absent***	Variant	***absent***	***absent***	None
3.9	Material rewards and avoidance of punishment	Variant	Typical	Variant	Variant	None
***Theme 4: Recipient of prosocial behaviours***						
4.1	Family	***absent***	Variant	Variant	***absent***	None
4.2	Friends	Variant	***absent***	Variant	Variant	None
4.3	Community	Variant	Variant	Variant	Variant	None
4.4	Strangers	Typical	Typical	Variant	General	Generational differences among Indonesian samples
4.5	Underprivileged or marginalised groups	Variant	***absent***	Variant	Typical	Cultural differences among younger adults

^*)^Occurrence of a theme within a group is “general” if applied to all participants, or all but one of the participants; “typical” if applied to more than half of the participants; “variant” if applied to less than half of the participants; and “absent” if not existed in all participants (Hill et al., 2012).

^**)^Group difference is evident with at least two categories differences (i.e., “absent” vs. “typical”; “variant” vs. “general”; and “absent” vs. “general”), as suggested by Ladany et al. (2012).

### Theme 1: layperson’s understanding of prosocial behaviour

This theme reflects participants’ understanding of what behaviour can be regarded as prosocial, the underlying processes of acting prosocially, and the changing nature of prosocial behaviour. Overall, all four groups show similar patterns when discussing the meaning of prosocial behaviour, the factors that contribute to prosocial acts, as well as the changing nature of prosociality. However, cultural differences among older adults were identified on the sub theme “How people decide to act prosocially”.

The first subtheme “What it means to be prosocial” explores participants’ plain understanding that prosocial behaviours require minimum effort such as performing low-risk help and displaying positive gestures towards others. One participant from Australian young adult group stated that prosocial behaviour was simply “*… just being considerate of other people around you … kind of being aware of other people and helping them in small capacities*” (Extract 1.1, AU-Y1F06). All groups listed effortless, spontaneous help that can be included in prosocial behaviours such as offering a seat at public transport, holding the door for other people, being tolerant of others, and being kind towards the others. Participants mentioned that displaying positive gestures is a form of prosocial signalling directed to give a positive experience for other people.

The second subtheme “How people decide to act prosocially” describes that people’s decision to perform prosocial behaviour may vary from an intuitive, automatic decision process to a deliberative, controlled process of thoughts. The way people decide to engage in prosocial behaviour was different between cultures for older groups. The Indonesian older adults generally discussed that potential risks associated with giving help such as trust to the recipient, personal sacrifice, and effectiveness of the help should be thoughtfully considered. One participant from the Indonesian older adults said that “*I would cautiously ask where my donation goes to. I only gave my donation to that organisation because I trust them, although I never had the chance to see the orphans by myself*” (Extract 1.2b, IN-O2F04). Due to the risk of fraud, charity donations should be given only through reputable organisations. On the other hand, Australian older generation mentioned that “*I was always naturally inclined to want to help people, anyway. I sort of outgrew that after thirty years, actually, but it was a framework that actually got me into the habit of thinking in that way*” (Extract 1.2a, AU-O1M01), suggesting that helping was a spontaneous, natural process of thoughts.

The third subtheme “Predictors of prosocial acts” explains two factors that predict prosocial behaviour: individual differences and socio-cultural context. Participants across groups variantly discussed different aspects of individual-level factors such as age (“*But the older you get, the more comfortable you are with making that final decision (of helping)*”) (Extract 1.3a, AU-Y1M07), gender, personality, self-efficacy, ideological belief (“*You went to Catholic school, so* you’d *know …* it’s *kind of the idea that everyone should have the same amount of wealth”*) (Extract 1.3c, AU-Y2F02), religion (“A*cts of charity and stuff like that are, maybe motivated, say, from a religious framework, but at least it’s being put out there. But I, I do agree that it’s a shame if it is the sole motivation*” (Extract 1.3b, AU-O1M01). Participants also mentioned socio-cultural factors that determine people to act prosocially such as parenting practices (“*With positive and pro-social behaviour, I feel it kind of does stem from a very young age, like, what you are brought up with*”) (Extract 1.3d, AU-Y2M01), and social responsibilities (“*My friend’s a nurse and she says, that if she is wearing her scrubs, there’s an incident, that she can likely get in trouble if she doesn’t help*”) (Extract 1.3e, AU-Y1F04).

The fourth subtheme “The changing nature of prosocial behaviour” captures participants’ understanding that forms, motives, and consequences of prosocial behaviours may change over time. For example, one participant form Australian young adults mentioned that “*Prosocial behaviour is being on social media, is posting, is … doing a hashtag, or something. Like, it is part… of conforming … and if you’re not seen to undertake those behaviours, you’re not seen as a social or normalised human being*” (Extract 1.4a, AU-Y2M01). The motive that drives people to act prosocially were not solely to benefit the others, but rather a mixture with personal motive to boost own morality, as participant from Australian young adult group said, “*Nothing is completely selfless. You know? Like, at the bottom of your heart, you’re still like, ‘That was a good thing I did. So, you may be doing it for that person in that instance, but, like, deeply it could be to consolidate your own morality somehow*” (Extract 1.4b, AU-Y1M07).

### Theme 2: types of prosocial behaviour

This is an overarching theme that covers different forms of prosocial behaviour. Five types were identified across groups as the subthemes. All groups confirm relatively similar patterns when identifying the five subthemes based on their experiences. Therefore, types of prosocial behaviour are identical across cultures and generations. The five subthemes are discussed below.

The subtheme “Altruistic prosocial behaviour” documents participants’ experiences in prosocial acts associated with sympathy. Participants mentioned prosocial acts such as giving charity donations (money, food, and goods), donating blood (“*but in terms of selflessness, I think the big one for me was giving blood regularly*”) (Extract 2.1, AU-Y1M02), and volunteering in assisting street children and underprivileged communities.

The subtheme “Anonymous prosocial behaviour” explains prosocial behaviour performed anonymously without knowing who received the help. Although this type of prosocial behaviour was not frequently mentioned, young Australians and Indonesians promoted online donations to reputable and well-established charity organisations. One participant from the Indonesian young adults mentioned, “*There are many crowd-funding platforms available online now. To me, I would prefer to give it online without them knowing my name. Only on the basis of urgency and severity*” (Extract 2.2, IN-Y2F05), suggesting anonymous preference for online donation.

The subtheme “Prosocial behaviour for compliance” refers to helping in response to others’ verbal or non-verbal requests. Participants frequently documented actions such as being kind to the consumer in work settings, doing household chores in a shared living space (“*I live in a house with, like, a few* housemates, *so, maybe just cleaning the kitchen or the bathroom if* I’m,* like, free, then I might as well do that, ‘cause, you know*, it’s *my living space, but* it’s also theirs. *So, if* it’s *something that I can do, then why* not just *do it?”*) (Extract 2.3, AU-Y1M07), responding to a friend’s request (e.g., being a designated driver) and giving non-serious help as a response to non-verbal situational cues (e.g., giving seats to the elderly).

The subtheme “Prosocial behaviour in dire circumstances” summarises helping performed under crises or emergencies. Helping in an emergency was less frequently reported across groups. However, participants mentioned such prosocial actions include helping an injured person in a traffic accident (“*It is just an automatic response, when I saw an accident, I am obliged to help*”) (Extract 2.4, IN-Y1M03), helping people with a mental health problem, and volunteering for natural disasters (earthquakes) by looking after young children at a temporary shelter.

The subtheme “Public prosocial behaviour” explains prosocial behaviour performed in front of the public to gain the approval and respect of others or enhance self-worth. Forms of prosocial behaviour performed in front of the public were frequently documented as participation in volunteering in local community events (“*My partner volunteers for the local community a bit at [a local arts festival] Darlington Arts Festival, um, and I volunteer at that, too. He does it a lot more but, um, I just do it on the weekend, um… We keep to things like, regularly*”) (Extract 2.5, AU-O1F08), picking up rubbish in public places, and contributing to a community’s social media group.

### Theme 3: prosocial motivations

This theme explains the motivational bases that drive people to engage in particular prosocial behaviour. Prosocial behaviour may stem from a range of altruistic to egoistic motives. Amongst nine subthemes documented from the participants, the subtheme “Empathic concern or sympathy” showed both generational and cultural differences. The subtheme “Reduce aversive or negative arousal” also showed cultural differences among younger adult groups.

The first subtheme “Empathic concern or sympathy” refers to motives associated with the ability to aim attention and responses towards others’ emotions and needs. Participants variantly discussed that sympathy and concern towards others motivated prosocial acts such that cultural and generational differences were identified in this subtheme. Cultural difference was observed in older generations, as the Australian older group discussed more frequently that their prosocial acts were based on empathy than the Indonesian older groups. Perspective-taking was more frequently discussed when deciding to help other people in need such as relating the current situation with personal experience, imagining that the person being helped was a family member, or putting themselves being at the same situation. One participant from Australian older group confirmed “*That’s about having empathy, too, I think, ‘cause you go, “Oh I know how I would feel”, you know, if that were me, yeah. ((crosstalk agreeing the statement)*” (Extract 3.1a, AU-O1F08). Generational differences were noted in Indonesian participants as Indonesian younger group generally mentioned helping in crisis was motivated by concern for others’ need for safety (“*I was thinking it was not safe for the victim being in the middle of the road, so I stopped and walked him to a safe corner. Things like that were driven by the reason that as human beings, we help each other. Kind of morally responsible because we are social beings*”) (Extract 3.1b, IN-Y1M03). Interestingly, the Indonesian older generation rarely discussed the prevalence of concern towards others as their prosocial motive within the groups.

The subtheme “Adherence to internalised principles” reflects participants’ motives attached to moral principles such as fairness and justice. For example, one participant from Indonesian young adult generation said, “*There is also a program for Indonesian children. It covers education for children in remote, almost isolated areas. I was self- funded to volunteer in this program. So, we voluntarily assigned ourselves there. Living with the locals for about two weeks or a month. I feel addicted to this… I feel happy seeing new people from different parts of the world. It is like… I was dictated to give something back to our environment*” (Extract 3.2, IN-Y2F05), suggesting that volunteering was driven by the desire to give back kindness to the community.

The subtheme “Adherence to social norms” explains prosocial motives associated with the desire to act following society’s norms. For example, participant from Australian younger generation said, “*I think there are things that I would do in some circumstances but not others, not necessarily because I wouldn’t be willing to, but because social norms dictate that it’s not really appropriate*” (Extract 3.3, AU-Y1F01).

The subtheme “Emphatic joy and sustaining positive emotion” explains motives related to the desire to maintain pre-existing positive emotions when other’s needs were met. Prosocial behaviour performed for the reason of maintaining positive emotions such as gratitude, satisfaction, pride, and happiness was typically reported in Australian groups (“*But there’s also a big sense of, um, you know, of pride, I guess… I don’t know the person who’s going to receive the blood. I have zero contact, but there is still a sense of, yeah, pride, I guess, that comes with it. I understand that I, right now, I’m in a better spot than you might be, complete stranger. Here’s what I can do to help*”) (Extract 3.4, AU-Y1M02). This confirms that helping was performed because it brought positivity to the giver.

The subtheme “Social relatedness” reflects participants’ motives for social interaction and belongingness with others, as one participant from Australian older generation mentioned, “*But I think most of us are motivated by wanting to belong to something, whichever group it is that we wanna belong to*” (Extract 3.5, AU-O1F03).

The subtheme “Goal completion and increased feelings of competence” describes participants’ desire to strive for mastery and enhance personal competence. Across Australia and Indonesia, younger generation groups typically reported that prosocial behaviour was aimed to improve personal competencies. For example, participant from older Australian group mentioned that “*I think a few people like to volunteer because it’ll look good on their resume as well. So, it’s, um, like you said it’s helping them to up-skill and develop. Um, also it benefits you in return as well because it can help you get a job, once you graduate*” (Extract 3.6, AU-O1F04).

The subtheme “Reduce aversive or negative arousal” explains motives to reduce participants’ distress or alleviate guilt. Motives related to reducing negative arousal appeared culturally different in younger generations. The Australian young groups mentioned that prosocial behaviour was evoked by the desire to reduce negative emotions. However, these motives did not present in all Indonesian groups. One participant from the Australian younger group described, “*I wouldn’t see myself as a good friend if I didn’t respond to that kind of hurt, you know, by offering support. Um, but I guess as well, like, you could see it as a bit selfish in other ways because, like, it reduced my distress by knowing that I had reduced his*” (Extract 3.7, AU-Y1F01), explaining that helping was performed because the helper felt someone’s pain as the pain in him/herself, and therefore, providing help was intended to soothe his/her own’s pain.

The subtheme “Social approval and reputation-related rewards” relates to motives associated with the desire to obtain acceptance and acknowledgement from the other. One participant from Australian younger group stated, “*I mean, like, I know it doesn’t, like, directly benefit me, but when I do something nice for, like, a random person, I kind of hope that they go home and go, ‘Oh, this nice, like, this lady, like, you know, helped me do this’. Or, ‘She did this for me’. And then that kind of makes me feel better… It benefits me because I feel like I’ve done, like, a good thing and they get to go home and tell their family about it*” (Extract 3.8, AU-Y1F06).

The subtheme “Material rewards and avoidance of punishment” reflects participants’ expectation to obtain something in return from the recipient. For example, participant from Australian young adult mentioned, “*If I donated blood or gave money or something, and that blood ended up, you know, being contaminated or my money was wasted on something, I don’t know, I feel like I would take… I would be much more disappointed in that. I feel like the more personal sacrifice your kindness comes at, the more you care about the outcome of it. Does that make sense?”* (Extract 3.9, AU-Y2F02).

### Theme 4: recipient of prosocial behaviour

This theme reflects recipients of prosocial behaviour. All cultures and generations similarly mentioned family, friends, and community as the recipient of prosocial behaviour. However, the groups showed differences when discussing strangers and underprivileged groups as the target of prosocial acts.

The subtheme “Family” describes the inclusion of immediate family as the primary receiver of prosocial behaviour. All group mentioned that family is the main recipient of prosocial behaviour (“*I was thinking, um, it depends on how I know the person and how much help I’m willing to give them, because there’s, like, random acts of kindness that you can do. There’s, like, standing up on the bus or opening the door for a stranger. Um, but in terms of like favours, I’m more likely to do, like, a significant favour for like a family [member] or a friend*” (Extract 4.1, AU-Y1F04).

The subtheme “Friends” includes friends and co-workers as recipients of help. Across cultures and generations, friends or co-workers were mentioned as the person to whom prosocial behaviour was usually directed to (“*Usually, they need my help for tax report issues. I know it should be their responsibility, but at least 20% of my colleagues needed me to assist them*”) (Extract 4.2, IN-O1F03).

The subtheme “Community” refers to society members whom participants feel attached. Examples of community-directed prosocial acts were supporting community art events, donating goods to the local community, and volunteering in community events (“*Oh yes, yes. Um, so my main focus in both my professional life and my personal life is supporting people with cancer, and so some of that I do as a paid role, but a lot of it I do as a volunteer and I get involved in community events like the [local fun run]*”) (Extract 4.3, AU-O1F07).

The subtheme “Strangers” refers to strangers as the recipient of prosocial behaviour, which was frequently mentioned, particularly in young adult groups. Generational differences were reported in Indonesian groups. Compared with the older generation, Indonesian young adult participants generally mentioned that their prosocial acts more likely directed to strangers rather than to people they feel familiar with (”*Compared to my Mum’s generation… She often asked me why I gave online donations. ‘Why did you give it to strangers and not to our relatives?’ I think because if I gave it to people I have familiar with, I would expect them to help me back in return, someday … and there would be a risk that it won’t be anonymous?*” (Extract 4.4, IN-Y2F01). In Indonesian older adult groups, directing prosocial acts to strangers were less likely discussed.

The subtheme “Underprivileged or marginalised groups” refers to disadvantaged communities such as street children, homeless people, people living in isolated areas, or victims of a natural disaster. This type of helping differed between the young adult groups in that it was typical for Indonesian young adults but was absent in Australian young adults. One participant from Australian young adults group mentioned that “*I think, you know, even if walking past you see somebody who looks like they’re homeless, it doesn’t mean that you would go out and buy them a meal, because you don’t want to feel like you’re stepping into, you know, their space*” (Extract 4.5, AU-Y1F03).

## Discussion

This focus group study explored cultural and generational understanding of prosocial behaviour practices with a series of focus group conducted in Australia and Indonesia. Informed by the theory of social change and the perspective of cultural dynamics, this study compared two distinctive cultural samples across generations that presumably experienced different pathways of socio-cultural changes. Overall, while meaning and forms of prosocial behaviour were in many ways similar across cultures and generations, cultural and generational differences were documented in some subthemes on prosocial conceptualisation, motivation, and recipients.

### Similarities across cultures and generations

Participants shared a similar understanding of prosocial behaviour that it can be seen as a simple positive behaviour directed at other people (subtheme 1.1). Participants across cultures and generations similarly mentioned a range of underlying factors of prosocial behaviour (subtheme 1.3). In subtheme 1.4, participants recognised the changing nature of prosocial behaviours, noting that it may change in terms of forms, motives, and consequences from time to time.

Types of prosocial behaviour were described similarly across groups (theme 2). Participants recalled different forms of prosocial acts they may experience or observe, which may vary from altruistic (subtheme 2.1), anonymous (subtheme 2.2), compliance (subtheme 2.3), dire (subtheme 2.4), and public (subtheme 2.5) types of prosocial actions. These types of prosociality were similar to Carlo and Randall’s (2002) typology of prosocial behaviour in early adulthood. It appears that because all participants were within the age continuum that enables them to autonomously display such prosocial acts, all types of prosocial behaviour were relevant to discussion for all participants.

In theme 3, participants across cultures and generations commonly perceived that prosocial behaviour was directed by various motives. Participants described that prosocial acts may stem from the desire to comply with moral principles (subtheme 3.2) and society’s norms (subtheme 3.3). Participants also noted that motives such as maintaining positive emotion (subtheme 3.4), social relations (subtheme 3.5), personal competence (subtheme 3.6), reputation (subtheme 3.8), as well as obtaining material rewards (subtheme 3.9) as the potential drivers of prosocial behaviour.

In attempts to differentiate motives for prosocial behaviour, Eisenberg et al. (2016) propose various kinds of prosocial motivations in a continuum that reflects other-benefiting to self-benefiting orientation. In the current study, participants reported both altruism and egoism motives to engage in prosocial behaviour with some degree of prevalence (Theme 3). This finding suggests that across cultures and generations, prosocial behaviour was driven by both other- and self-orientation motives. This is consistent with the notion of dualistic motives of prosocial behaviour, where prosocial behaviour is regarded as performed by two selves: the self as an actor, which was motivated by idealistic moral values, and, the self as an executor, which was motivated by realistic selfish motives (Batson & Powell, 2003; Frimer et al., 2014). Therefore, all participants understand that performing prosocial acts may stem from both altruistic and egoistic motives.

In theme 4, participants similarly mentioned that prosocial behaviour was directed to family (subtheme 4.1), friends (subtheme 4.2), and community (subtheme 4.3). It is noteworthy that helping strangers was more frequently discussed within each group except for Indonesian older groups. Directing prosocial acts to people with familiarity and acquaintances might be perceived as the fulfilment of duties to show that individuals have internalised social expectations (Gherghel et al., 2020). As such, it may be the case that there is no need to raise concern to discuss prosocial behaviour directed at a person one knows since helping and caring for this person might be valued as a common behaviour that represents compliance with social norms and fulfilment of social obligations. In contrast, in a public forum like a focus group, discussing helping strangers can be potentially valued (i.e., to increase self-presentation in front of others). That is, participants might have judged that the action of helping strangers requires much effort to satisfy social expectations (McAuliffe et al., 2020) such that this topic might have been seen as worthwhile to discuss more frequently by the participants.

### Differences across cultures and generations

Between group differences were observed in the way people decide how to act prosocially (subtheme 1.2) in Indonesian and Australian older adult groups. Indonesian older adults were more frequently discussed risks associated with deliberately processed prosocial acts than Australian older adults. To the extent that the decision to act prosocially may involve two different processes of thinking: intuitive or deliberative (Rand & Epstein, 2014), one interpretation is that, for the Indonesian older generation, performing prosocial acts may not be intuitive but requires careful thinking and effort. The theory of individualism-collectivism posits that people from collectivistic backgrounds perceived a greater obligation to respond to others in need than those from individualistic cultural backgrounds (Gherghel et al., 2020) such that performing prosocial acts may be perceived as responding to social obligations and valued as a highly moralized choice in collectivistic cultures. Therefore, for Indonesian older adults that may hold strong preferences towards collectivistic values, prosocial responses may be decided using careful consideration since it represents moral choice rather than relying on intuitive speculations. Another difference observed between older adult groups was that the Australian older generation discussed empathic concern or sympathy motives more than the Indonesian older adults (subtheme 3.1). This finding is contrary to previous research that suggests higher collectivism countries are associated with a higher level of empathy (Chopik et al., 2017). Our interpretation is that Indonesian older adults may perceive prosocial actions as social obligations they had to fulfil to society rather than seeing prosocial actions as stemming from empathetic concern for others.

Between group differences in Indonesian and Australian younger generations were recorded in two subthemes: reduce aversive or negative arousal (subtheme 3.7) and directing prosocial acts to underprivileged or marginalised groups (subtheme 4.5). Australian young adults more frequently reported that prosocial acts were provoked by the intention to reduce negative arousals, such as reducing their own’s distress or eliminating worry and sadness. In Indonesian young adults, this topic was absent. One interpretation of this finding is that for Australian young adults, performing prosocial acts provoked by the concern to alleviate self-distress reflects an expression of individual choice, which may be seen as an indication of agentic motivations prevalent in individualistic societies (Gherghel et al., 2020). With respect to recipients of prosocial behaviours, Indonesian young adults reported helping directed to underprivileged or marginal groups such as homeless people, street children and geographically isolated communities. In Australian young adults, helping directed to this type of recipient was absent, possibly because helping less privileged communities could be perceived as overstepping the boundary (Extract 4.5).

The study observed within group differences only in Indonesian younger and older groups concerning two subthemes: empathic concern motives (subtheme 3.1) and strangers as the recipient of prosocial behaviour (subtheme 4.4). Compared to older adults, Indonesian young adults more frequently discussed that prosocial act reflecting empathic concern towards others (subtheme 3.1). The current data also suggests that Indonesian young adults were more likely to direct their prosocial acts to strangers (subtheme 4.4). These findings may suggest that for Indonesian generations, conceptualisation and practices of prosocial behaviour may not as congruent as found in Australian societies.

Theoretically driven, the direction of social change into global increased in individualism (Greenfield, 2016; Santos et al., 2017) offers an explanation that changing value preferences may occur in Indonesia and Australia. Findings on group similarities across cultural and generational groups in Indonesia and Australia may indicate that some attributes of prosociality (i.e., types) are broadly evident across societies. Differences between older and younger generations found in Australia and Indonesia suggest that cultural diversity may present in terms of the way prosocial behaviour is understood and practices by each cultural domain. Lastly, within group differences found only in Indonesian participants may indicate that the meaning and practices of prosocial behaviour in Indonesian society may not as congruent as found in Australia.

### Limitations and direction for future research

In this focus group study, increased social desirability among participants may present, such that it may affect the way people respond to each other. Particularly in Australia, some participants were recruited from the university research participant pool, such that there is a possibility of familiarity with other participants that may induce participants to give responses that conform with desired moral characters within a group (McAuliffe et al., 2020). Since the study attempted to gain insight into how people conceptualise different aspects of prosociality through dialogue, the risk of social desirability bias was unavoidable (Gilbert et al., 2019). Prosocial behaviour is a desirable behaviour expected in every culture and society (Twenge et al., 2007) such that participants may feel the topic worth presented to obtain public recognition rather than to express the actual experiences they may have. Future studies may be directed to specify the inquiry into actual personal experiences when performing prosocial acts. Since female participants were over-represented in this study, there is a possibility that the prosocial behaviour reported here may reflect those more accessible to the female gender. Future research should attempt to minimise social desirability and gender imbalance by implementing a different approach to participant recruitment.

The current study indicates generational differences found in Indonesian groups in terms of prosocial motives and recipients. While Callaghan and Corbit (2018) noted that only a few studies examine the psychological and cultural mechanisms of similarity or diversity of prosocial behaviour, this focus group study was not directed to provide an explanation of how differences and similarities across generations may occur. Future research is required to investigate the way prosocial behaviour can be maintained or changed from time to time in a different cultural context.

## Conclusions

The current findings suggest that although the pattern of similarities was predominant across cultures and generations, differences in aspects of prosociality are evident and worth explaining. Cultural differences observed may reflect the fact that Australia and Indonesia hold different perspectives of prosocial behaviour. Further, generational differences were evident in Indonesian but not in Australian participants. This finding may be an indication that, for Indonesian generations, cultural information related to prosocial behaviour may not appear as similar as found in Australian generations. This finding may support the notation that changes in sociocultural contexts experienced by different societies may be associated with the way cultural information such as prosociality can be understood and practised over time.

## Data Availability

Ethical approval precludes the data being made available in a data repository. Specifically, the ethical approval specifies that all results are in aggregate form to maintain confidentiality and privacy and precludes individual level data being made publicly available. All aggregate data for this study are freely available and included in the paper.

## Appendix. Extract samples

**Table:**

Themes and Subthemes	Exemplar Extracts
**Theme 1: Layperson’s understanding of prosocial behaviour**	
1.1 What it means to be prosocial	*“I think even sometimes less than that. It’s just being considerate of other people around you. So, if you’ve got some extra time on a parking ticket, giving that to somebody because you don’t need it, or offering to stand because you don’t need to sit. Just kind of being aware of other people and helping them in small capacities”*. (Extract 1.1, AU-Y1F06)
1.2 how people decide to act prosocially	*“I guess I, I was always naturally inclined to want to help people, anyway. I sort of outgrew that after thirty years, actually, but it was a framework that actually got me into the habit of thinking in that way”*. (Extract 1.2a, AU-O1M01) *“I usually give donations for orphans to a charity organisation. A volunteer would come to me so I could drop some money on a regular basis. I would cautiously ask where my donation goes to. I only gave my donation to that organisation because I trust them, although I never had the chance to see the orphans by myself”*. (Extract 1.2b, IN-O2F04)
1.3 The predictors of prosocial acts	*“You start to form, like, more solid opinions and, you know, logic so you can start to form … well, at least something you think is logical, anyway, and makes sense to you. But when you’re younger, it’s harder to make that decision, to make a solid decision that you’re certain is the right thing to do. But the older you get, the more comfortable you are with making that final decision”*. (Extract 1.3a, AU-Y1M07) *“I think like, um, if people are motivated, say, by the religious thing or by anything like that, I’m not, I mean, and I’ll come back to it, but, um. I -it provides a framework for those who either won’t, would never have thought of it, or might not feel obliged to do certain things so that these say, uh, acts of charity and stuff like that are, maybe motivated, say, from a religious framework, but at least it’s being put out there. But I, I do agree that it’s a shame if it is the sole motivation”*. (Extract 1.3b, AU-O1M01) *“No, I’m sure, like, for me, guilt is a big part of it. Because, I… like, do you go… do you guys know about the universal distribution of goods? You went to Catholic school, so you’d know. Yeah. So, it’s kind of the idea that everyone should have the same amount of wealth. Um, and I don’t know if that was drilled into me or that was how it was, but I see myself as a rather well-off person, and I feel like it’s my responsibility, (laughs) this is whack, to distribute my wealth to people who are not necessarily as wealthy”*. (Extract 1.3c, AU-Y2F02) *“I think… I think with the positive… yeah, with positive and pro-social behaviour, I feel it kind of does stem from a very young age, like, what you are brought up with, I feel, especially with children. Especially as, like, when you’re looking at it from a psychological perspective we’re kind of… we’re kind of these pieces of dough when we’re younger, and, (laughs) like, like, or like play dough, and every, every experience, um, especially interacting with our parents and interacting with our family and our family in a cultural context, it kind of moulds the dough a bit more. Um, and influences our behaviour later on down… like, later on down the line, so, as an adult, as a teenager. So, subconsciously, we are kind of mimicking all those behaviours and kind of, well, we’re kind of… these super behaviours that we’re kind of drawing from, and all these learning experiences that we’ve had”* (Extract 1.3d, AU-Y2M01) *“My friend’s a nurse and she says that if she is wearing her scrubs, there’s an incident, that she can likely get in trouble if she doesn’t help … um, because if they see her scrubs, and she just keeps on going by, then she can … someone can complain about it. So, she was saying that she, like, just changes out of her uniform when she’s, going home”*. (Extract 1.3e, AU-Y1F04)
1.4 The changing nature of prosocial behaviour	*“I feel like prosocial behaviour is conforming to, is conforming to the social ideologies of the day. … Prosocial behaviour is being on social media, is posting, is … doing a hashtag, or something. Like, it is part… it is part of… it’s conforming. It’s… and if you’re not seen to undertake those behaviours, you’re not seen as a social or normalised human being”*. (Extract 1.4a, AU-Y2M01) *“Yeah, that’s kind of why I said relatively selfless because nothing is completely selfless. You know? Like, at the bottom of your heart, you’re still like, ‘That was a good thing I did. So, you may be doing it for that person in that instance, but, like, deeply it could be to consolidate your own morality somehow”*. (Extract 1.4b, AU-Y1M07) *“I feel like you might relate to this. I feel like when… with this netball thing that I’ve been telling you guys about, every single time I catch a glimpse of any committee member, they just bombard me with, like, “Post this, do this, do that, we need this doing”, and instead of being like, “No, I have three uni assessments to do this week, I have absolutely zero time” I just fill my plate ‘til it’s literally overflowing. I’m like, “No, I can get it done”. And then I forgo my sleep, (laughs) my well-being, my uni grades. All goes out the window just to help people”*. (Extract 1.4c, AU-Y2F04)
**Theme 2: Types of prosocial behaviour**	
2.1 Altruistic prosocial behaviour	*“but in terms of selflessness, I think the big one for me was giving blood regularly”*. (Extract 2.1, AU-Y1M02)
2.2 Anonymous prosocial behaviour	*“There are many crowd-funding platforms available online now. To me, I would prefer to give it online without them knowing my name. Only on the basis of urgency and severity”*. (Extract 2.2, IN-Y2F05)
2.3 Prosocial behaviour for compliance	*“Well, maybe, because I live in a house with, like, a few housemates, so, maybe just cleaning the kitchen or the bathroom if I’m, like, free, then I might as well do that, ‘cause, you know, it’s my living space, but it’s also theirs. So, if it’s something that I can do, then why not just do it? You know?”* (Extract 2.3, AU-Y1M07)
2.4 Prosocial behaviour in dire circumstances	*“One example that I often did was when I saw people injured in a traffic accident. In that situation, I spontaneously stopped and directly helped the victim. It is just an automatic response, when I saw an accident, I am obliged to help”*. (Extract 2.4, IN-Y1M03)
2.5 Public prosocial behaviour	*“My partner volunteers for the local community a bit at [a local arts festival] Darlington Arts Festival, um, and I volunteer at that, too. He does it a lot more but, um, I just do it on the weekend, um… We keep to things like, regularly”*. (Extract 2.5, AU-O1F08)
**Theme 3: Prosocial motivations**	
3.1 Empathic concern or sympathy	*“That’s about having empathy, too, I think, ‘cause you go, “Oh I know how I would feel”, you know, if that were me, yeah. (crosstalk)”* (Extract 3.1a, AU-O1F08) *“I was thinking it was not safe for the victim being in the middle of the road, so I stopped and walked him to a safe corner. Things like that were driven by the reason that as human beings, we help each other. Kind of morally responsible because we are social beings”*. (Extract 3.1b, IN-Y1M03)
3.2 Adherence to internalised principles	*“There… there is also a program for Indonesian children. It covers education for children in remote, almost isolated areas. I was self- funded to volunteer in this program. So, we voluntarily assigned ourselves there. Living with the locals for about two weeks or a month. I feel addicted to this… I feel happy seeing new people from different parts of the world. It is like… I was dictated to give something back to our environment”*. (Extract 3.2, IN-Y2F05)
3.3 Adherence to social norms	*“I think there are things that I would do in some circumstances but not others, not necessarily because I wouldn’t be willing to, but because social norms dictate that it’s not really appropriate”*. (Extract 3.3, AU-Y1F01)
3.4 Empathic joy and sustaining positive emotion	*“. and I was sitting down. But there’s also a big sense of, um, you know, of pride, I guess. It’s not really … I don’t know the person who’s going to receive the blood. I have zero contact, but there is still a sense of, yeah, pride, I guess, that comes with it. I understand that I, right now, I’m in a better spot than you might be, complete stranger. Here’s what I can do to help”*. (Extract 3.4, AU-Y1M02)
3.5 Social relatedness	*“So, um, but then there’s a different perspective to that, you’ve got the group that wants to be the in, in a group so it’s a … But I think most of us are motivated by wanting to belong to something, whichever group it is that we wanna belong to”*. (Extract 3.5, AU-O1F03)
3.6 Goal completion and increased feelings of competence	*“I think with, um, volunteering, I think a few people like to volunteer because it’ll look good on their resume as well. So, it’s, um, like you said it’s helping them to up-skill and develop. Um, also it benefits you in return as well because it can help you get a job, once you graduate”*. (Extract 3.6, AU-O1F04)
3.7 Reduce aversive or negative arousal	*“Like, I think as a friend that’s … I wouldn’t see myself as a good friend if I didn’t respond to that kind of hurt, you know, by offering support. Um, but I guess as well, like, you could see it as a bit selfish in other ways because, like, it reduced my distress by knowing that I had reduced his”*. (Extract 3.7, AU-Y1F01)
3.8 Social approval and reputation-related rewards	*“I mean, like, I know it doesn’t, like, directly benefit me, but when I do something nice for, like, a random person, I kind of hope that they go home and go, ‘Oh, this nice, like, this lady, like, you know, helped me do this’. Or, ‘She did this for me’. And then that kind of makes me feel better… It benefits me because I feel like I’ve done, like, a good thing and they get to go home and tell their family about it”*. (Extract 3.8, AU-Y1F06)
3.9 Material rewards and avoidance of punishment	*“I feel like circling back to what outcomes we would want, personally I feel like if I give a smile to someone, or, you know, have a nice conversation with someone and they walk away and don’t really care for that conversation at all, I probably personally wouldn’t feel like that’s any loss off my back. Yet if I donated blood or gave money or something, and that blood ended up, you know, being contaminated or my money was wasted on something, I don’t know, I feel like I would take… I would be much more disappointed in that. I feel like the more personal sacrifice your kindness comes at, the more you care about the outcome of it. Does that make sense?”* (Extract 3.9, AU-Y2F02)
**Theme 4: Recipient of prosocial behaviours**	
4.1 Family	*“I was thinking, um, it depends on how I know the person and how much help I’m willing to give them, because there’s, like, random acts of kindness that you can do. There’s, like, standing up on the bus or opening the door for a stranger. Um, but in terms of like favours, I’m more likely to do, like, a significant favour for like a family [member]or a friend”*. (Extract 4.1, AU-Y1F04)
4.2 Friends	*“Usually, they need my help for tax report issues. I know it should be their responsibility, but at least 20% of my colleagues needed me to assist them”*. (Extract 4.2, IN-O1F03)
4.3 Community	*“Oh yes, yes. Um, so my main focus in both my professional life and my personal life is supporting people with cancer, and so some of that I do as a paid role, but a lot of it I do as a volunteer and I get involved in community events like the [local fun run], (crosstalk) my training has been really terrible this year (laughter), but I’m still doing it, and I’m hoping people will (crosstalk) um so that’s probably the main, kind of, obvious charitable work”*. (Extract 4.3, AU-O1F07)
4.4 Strangers	*“Compared to my Mum’s generation…She often asked me why I gave online donations. ‘Why did you give it to strangers and not to our relatives?’ I think because if I gave it to people I have familiar with, I would expect them to help me back in return, someday… and there would be a risk that it won’t be anonymous?”* (Extract 4.4, IN-Y2F01)
4.5 Underprivileged or marginalised groups	*“I think, you know, even if walking past you see somebody who looks like they’re homeless, it doesn’t mean that you would go out and buy them a meal, because you don’t want to feel like you’re stepping into, you know, their space*”. (Extract 4.5, AU-Y1F03)
